# Single α-particle irradiation permits real-time visualization of RNF8 accumulation at DNA damaged sites

**DOI:** 10.1038/srep41764

**Published:** 2017-01-31

**Authors:** Giovanna Muggiolu, Michal Pomorski, Gérard Claverie, Guillaume Berthet, Christine Mer-Calfati, Samuel Saada, Guillaume Devès, Marina Simon, Hervé Seznec, Philippe Barberet

**Affiliations:** 1 Université de Bordeaux, Centre d’Etudes Nucléaires Bordeaux Gradignan (CENBG), Chemin du Solarium, 33175 Gradignan, France; 2 CNRS, UMR5797, Centre d’Etudes Nucléaires Bordeaux Gradignan (CENBG), Chemin du Solarium, 33175 Gradignan, France; 3 CEA-LIST, Diamond Sensors Laboratory, Gif-sur-Yvette F-91191, France

## Abstract

As well as being a significant source of environmental radiation exposure, α-particles are increasingly considered for use in targeted radiation therapy. A better understanding of α-particle induced damage at the DNA scale can be achieved by following their tracks in real-time in targeted living cells. Focused α-particle microbeams can facilitate this but, due to their low energy (up to a few MeV) and limited range, α-particles detection, delivery, and follow-up observations of radiation-induced damage remain difficult. In this study, we developed a thin Boron-doped Nano-Crystalline Diamond membrane that allows reliable single α-particles detection and single cell irradiation with negligible beam scattering. The radiation-induced responses of single 3 MeV α-particles delivered with focused microbeam are visualized *in situ* over thirty minutes after irradiation by the accumulation of the GFP-tagged RNF8 protein at DNA damaged sites.

Every day humans are exposed to ionizing radiation from natural, industrial and medical sources. A significant part of the natural background radiation exposure is caused by α-particles from the inhalation of radon gas^1^. In addition, α-particles are increasingly considered in medical applications, such as targeted radiation therapy, where α-emitting radionuclides are specifically localised to deliver a cytotoxic radiation dose to cancerous tissues, while sparing surrounding healthy tissues^2^,^3^,^4^. When traversing cells, α-particles induce clustered molecular damages along their tracks as a function of their Linear Energy Transfer (LET) which is the energy transferred per unit length by ionizing radiation along its path. These clustered DNA damages, involving single and double strand breaks, occur when two or more lesions take place within one or two helical turns of the DNA strand. These can be designed as complex DNA damages and they are particularly deleterious because more difficult to repair^5^,^6^. Therefore, potential health effects resulting from α-particles exposure continue to be the focus of numerous studies^7^,^8^,^9^,^10^,^11^,^12^,^13^. Understanding cellular responses to complex DNA damages specifically induced by α-particles is of particular importance and requires specific tools that allow the selective irradiation of single cells and follow-up observations of induced damage *via* dedicated biological markers (DNA damage signalling, DNA repair protein,..).

This can be achieved by exposing living cells to radioactive sources alongside immuno-detection^8^ or fluorescence live cell imaging^9^. However using this approach, the number of traversals at the single cell level cannot be precisely controlled and the correlation of the particle traversals with observed biological responses relies on the retrospective observations made by nuclear track detectors^14^,^15^,^16^,^17^,^18^,^19^.

Alternatively, charged-particle microbeams can target living cells with single charged particles and have been used extensively to study various biological endpoints at low doses^20^,^21^. Modern end-stations, equipped with fluorescence time-lapse imaging, provide the opportunity to visualize and quantify in real-time the early radiation-induced cellular response. Using these techniques, studies of DNA damage and repair kinetics^22^,^23^,^24^, DNA double strand breaks diffusion characteristics^25^, and calcium alteration due to heavy-ions^26^ have been conducted. Up to now however, most studies have been performed using energetic heavy ions with high-LET, such as a carbon ions or heavier particles with LET ranging from a few hundred to several thousand keV/μm. Electrostatic accelerators delivering Helium ions allow to mimic perfectly the effects of α-particles (in the following, He ions will be designated as α-particles for clarity). Despite these accelerators are relatively common, investigations using MeV α-particles remain scarce. Indeed, delivering single α-particles requires the insertion of a thin detector in the beam path upstream of the sample. However, due to the limited range of α-particles in matter (a few tens of micrometres), this is difficult to accomplish without significantly altering the microbeam energy and size. The method used on several microbeam facilities, consisting of removing the cell nutrient medium and detecting the particles downstream the sample^27^,^28^, is not applicable when performing time-lapse imaging online over long periods during and after irradiation.

Several types of thin transmission detectors have been developed to achieve this goal: thin plastic scintillators coupled to photomultiplier tubes^29^,^30^, thin silicon detectors^31^,^32^ and gas detectors^33^. More suitably, thin diamond membranes have shown very promising features for efficiently detecting single charged particles^34^,^35^,^36^. Nevertheless, all the detectors mentioned previously are usually a few micrometres thick and cannot be used to detect relatively low energy ions, such as MeV Helium ions delivered by conventional electrostatic accelerators.

In addition to the design of a specific detection system, visualizing and following the induced response of a single α-particle in living cells requires appropriate biological markers^9^. Several proteins are considered to be involved in the different steps of recognition, signalling and repair of DNA damages^37^. The activation of the MRN complex (including MRE11, RAD50 and NBS1 proteins), together with the recruitment to damaged areas of MDC1 and 53BP1 proteins, are the most studied mechanisms involved in DNA damage response^38^. These biomarkers tagged with fluorescent proteins, have allowed several laboratories to visualize the impact of single ion tracks in living cells^9^,^23^,^24^,^39^. However, a myriad of proteins acts during responses to DNA lesions, and most of them are primarily investigated with laser microbeams. Of particular importance, the discovery and characterization of the ubiquitin ligase RNF8 identify it as a key regulator of the rapid assembly of DNA repair complexes to DNA damage^40^,^41^,^42^. Several studies showed that RNF8 colocalizes with the DNA-damage marker γH2AX, and the strong collaboration with MDC1, NBS1 and 53BP1 proteins implies its critical role in the response to DNA damage^40^,^41^,^42^. More recently, its action mechanism and role in promoting DSB-associated chromatin ubiquitylation was shown^43^,^44^ and the rapid dynamics was revealed with photo-bleaching experiments^45^,^46^.

Here, we report the development of a thin α-particle detector based on secondary electron emission. This method, initially developed for energetic heavy ions^35^, is presently not used in routine on microbeam irradiators. We revisited and improved this approach to provide efficient Helium ions detection (as these ions perfectly mimic the effect of α-particles). It relies on an ultra-thin free-standing Boron-doped Nano-Crystalline Diamond film (BNCD) of a few hundred nanometres in thickness. By collecting the secondary electrons (SE) emitted from the surface, this active vacuum window allows simultaneous extraction in air and detection of single α-particles with minimal alteration of the microbeam energy and without interfering considerably with the α-particle track trajectory. This technical development allows us to irradiate a stable cell line expressing the GFP-tagged RNF8 protein that accumulates at DNA damage sites, forming the so-called ionizing radiation induced-foci (IRIF). Combining detection and irradiation we can visualize, for the first time, one α-particle track within a few minutes after irradiation in living cells and follow in real-time the fluorescent signal evolution.

## Results

### Characterisation of the BNCD membranes

**Figure 1:**
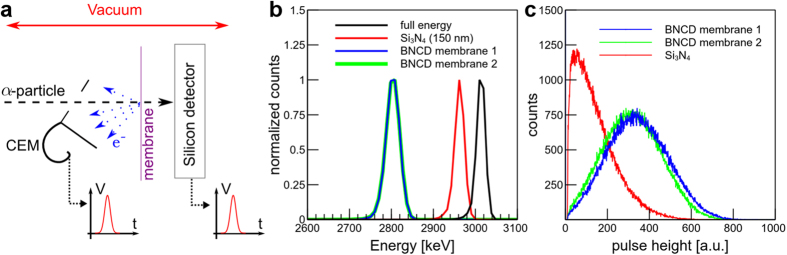
Characterization of the BNCD membranes. (**a**) Experimental set-up. The BNCD membranes were tested under vacuum for electron emission and thickness measurements. Electrical pulses induced by α-particle hits were acquired simultaneously from both CEM (channel electron multiplier) and thick silicon detectors. (**b**) Impact of the BNCD membranes on the transmitted energy. The red spectrum shows the energy transmitted through a 150 nm thick commercially available Si_3_N_4_ window. The two BNCD membranes (blue and green spectra) induce the same energy loss, thus the overlapped spectra. The black curve shows the beam energy without any material in its path. When passing through the BNCD membranes, an average energy loss of 200 keV is measured. (**c**) Channeltron pulse height spectra. The channeltron output signal is clearly separated from background for both BNCD membranes. The red curve shows the spectrum obtained on a Si_3_N_4_ window without any coating.

The energy of α-particles transmitted through the BNCD membrane was measured using the experimental set-up depicted on Fig. 1a. It provides a simultaneous measurement of the electrons emitted from the BNCD surface and of the energy of the particles transmitted through the membrane. Figure 1b shows transmission spectra obtained with the silicon detector positioned downstream the BNCD samples. A mean energy loss of 200 keV through BNCD and its Si_3_N_4_ supporting layer was measured on several membranes. Using the SRIM software (Stopping and Range of Ions in Matter)^47^, we estimated a mean thickness of 400 nm for the BNCD layer. Figure 1c shows channeltron pulse height spectra for two representative BNCD membranes. Compared to native Si_3_N_4_ windows, the channeltron signal is clearly amplified indicating an enhanced SE yield. When using a BNCD layer, every single pulse can be unambiguously separated from the background. Dark counts, i.e. counts registered without the α-particle beam, were below 5 s^−1^ in all cases. By comparing the number of counts registered in the spectra from Fig. 1b and c, the detection efficiency of the secondary electron detector was measured. In all cases, the difference in the number of counts on both detectors was lower than 0.2%. A detection efficiency of 100% was thus obtained and reproduced for several BNCD membranes.

The scanning capabilities of the microbeam allowed also maps to be created for both signals. The analysis of the transmitted energy shows that, in a scanned area of 400 × 400 μm, the maximum difference in energy loss from one beam position to the other is 30 keV (Supplementary Fig. S1). Maps obtained from the secondary electron detection over the same surface are also very homogeneous indicating a position-independent detection efficiency (Supplementary Fig. S2).

### Irradiations of track detectors

**Figure 2:**
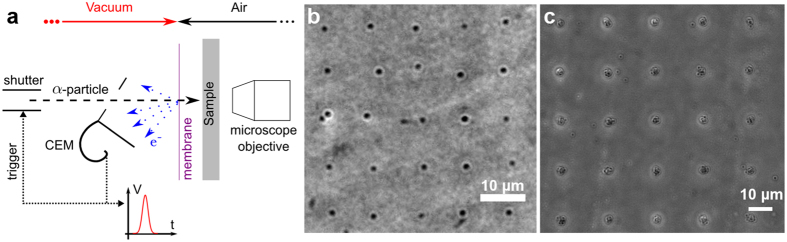
CR39 track detectors irradiated with counted α-particles. (**a**) The BNCD membranes were validated as vacuum windows by irradiating track detectors or cells with single α-particles in air. Pulses from the CEM were used to trigger the beam shutter when the required number of hits was reached. (**b**) Single particle irradiation in a regular pattern every 10 μm. Black dots correspond to etched tracks. (**c**) 10 particles delivered every 20 μm. All particles are delivered in a 5-μm diameter circle.

Irradiating solid-state track detectors, such as CR39, is a reliable way to assess simultaneously the ability of the system to deliver single α-particles and the influence of the membrane on the beam spot size. Figure 2 shows the results obtained in CR39 track detectors irradiated with one or more α-particles delivered in regular patterns. In that case, the BNCD membrane was used as a vacuum window and the beam was extracted in air before reaching the CR39 (Fig. 2a). From these measurements, we confirm that one single α-particle can be delivered at every beam position (Fig. 2b) and that the beam spot size is not degraded due to the angular scattering in the BNCD membrane. Delivering 10 α-particles per spot, as shown in Fig. 2c, we estimated that all particles are delivered in a circle of 5 μm in diameter. This corresponds to a beam FWHM (Full Width at Half Maximum) of about 2 μm.

### Induction of GFP-RNF8 recruitment to DNA damages induced by α-particles

**Figure 3:**
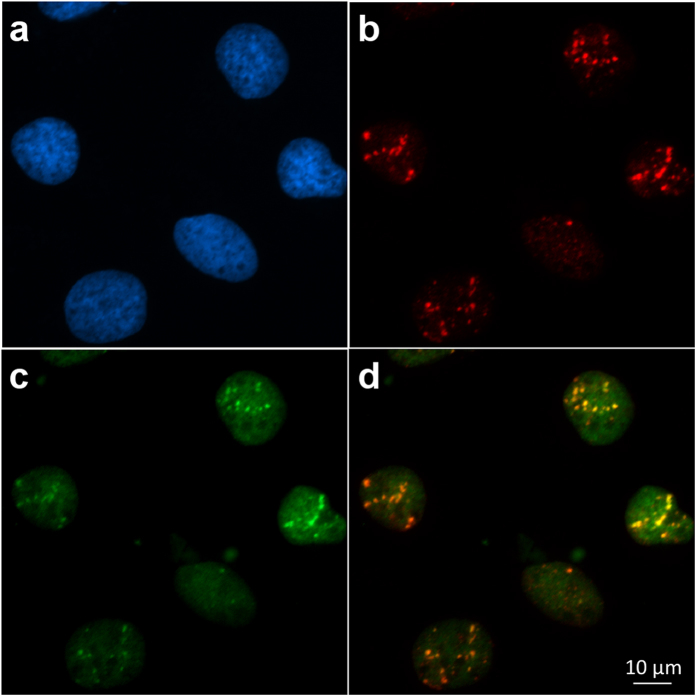
Induction GFP-RNF8 and γH2AX IRIF in cells irradiated with random α-particles. (**a**) Hoechst^33342^ staining reveals the nuclear chromatin. (**b**) IRIF are visualized with γH2AX immuno-detection in fixed cells exposed to ^239^Pu source. (**c**) GFP-RNF8 is re-localized to the DNA damaged areas, and (**d**) the merged image shows the overlap of GFP-RNF8 and γH2AX 30 minutes after irradiation.

Phosphorylation of the H2AX histone (serine 139), so called γH2AX, is known to be associated with DNA damages^48^. Therefore, the capability of GFP-RNF8 to be recruited in distinct IRIF, at DNA damaged sites induced by single α-particles, was assessed by the colocalization with immuno-detected γH2AX. Figure 3 shows cells exposed to a random α-particles irradiation from ^239^Pu source. γH2AX signal (Fig. 3b), obtained in cells fixed 30 min after irradiation is localized along α-particles tracks. The accumulation of GFP-RNF8 is also observed at damaged sites within the first hour following irradiation (Fig. 3c). Co-localization of GFP-RNF8 and γH2AX is depicted on the merged image confirming the presence of both proteins at damaged areas (Fig. 3d).

### RNF8 induction and accumulation dynamics at localized DNA damaged sites

**Figure 4:**
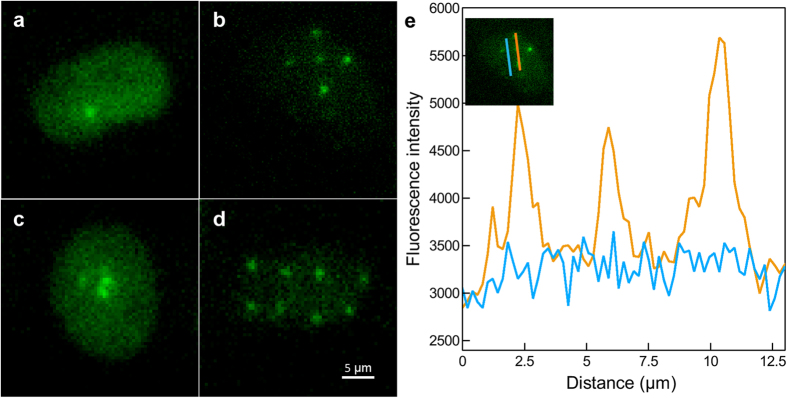
Heterogeneity of GFP-RNF8 response in cell nuclei irradiated with different patterns. Cells are irradiated with (**a**) one α-particle, (**b**) 5 α-particles distributed on a cross pattern. The targeted positions are separated by 4 μm. (**c**) 10 α-particles focalized on one position, and (**d**) regular pattern scanned over the whole microscope field of view. 8 α-particles induced-foci are distributed in the nucleus. (**e**) Fluorescence intensities measured along the two lines drawn in the inset plotted against their length. The blue line shows the constant nuclear fluorescence background; the orange line shows three peaks corresponding to the IRIF. Three different fluorescence intensities are observed suggesting an inhomogeneous chromatin density.

The main feature of microbeam is the ability to target and irradiate single cells with a precise number of α-particles distributed in a regular pattern. Here we achieved the detection of single α-particles using the BNCD membranes previously described. Figure 4 shows representative patterns illustrating the irradiation capabilities of the microbeam used. Single α-particles induced-foci are clearly visible at the irradiated sites (Fig. 4a, b and d). Targeted points separated by 4 μm are easily discernible confirming a beam resolution below 2 μm. Figure 4c shows the focus induced by 10 α-particles, whose diameter corresponds to the beam size. The intensity profiles of example GFP-RNF8 IRIFs 30 minutes after irradiation are illustrated in Fig. 4e. Even if the three IRIFs, indicated by green peaks, are each induced by exactly one α-particle, and thus by the same energy deposit, different fluorescence intensities are observed. These varying intensities could be explained by different chromatin configuration (hetero- and euchromatin) and densities, or by the presence of nucleolar compartments in the irradiated area. The brightest IRIF shows an increased fluorescence signal about two times greater than the background fluorescence intensity (Fig. 4e, blue line). The different IRIF distributed at various places in cell nucleus showed similar sizes of about 1 μm FWHM.

**Figure 5:**
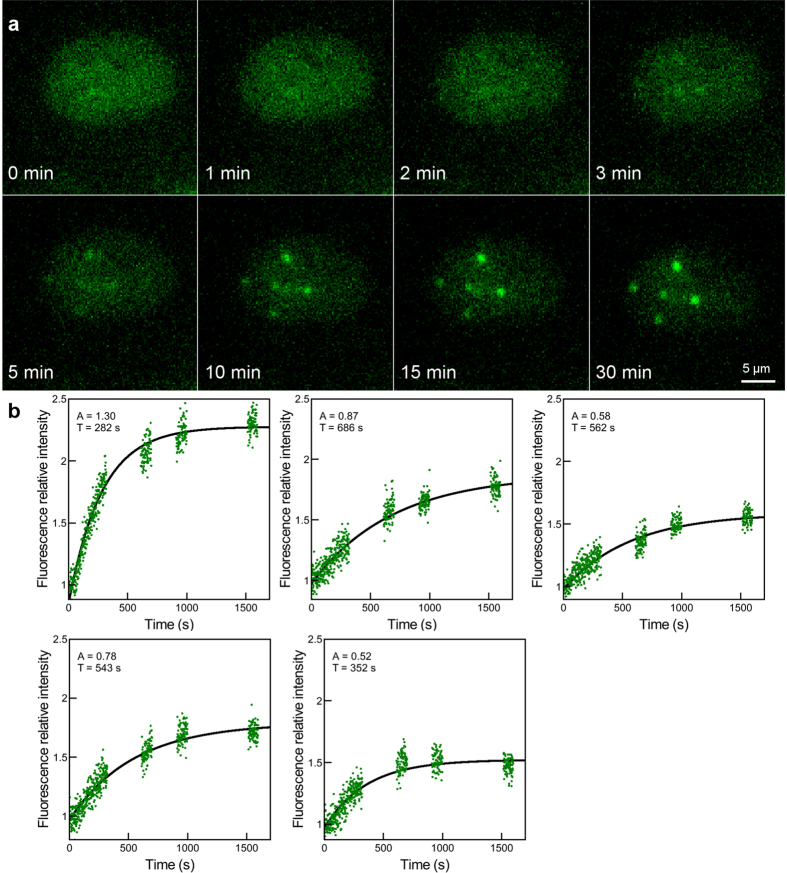
Time-lapse imaging of GFP-RNF8 in a cell nucleus irradiated with single α-particles. (**a**) The cell nucleus is targeted and irradiated at time 0 with a single α-particle per point on a cross pattern, each point separated by 4 μm. The re-localization of GFP-RNF8 is observed over 30 min following irradiation and selected time points are shown. (**b**) Kinetics curves of GFP-RNF8 corresponding to each IRIF obtained from experimetal data. Experimental curves (green points) are normalized and fit (black line) with a model for the first-order response. The intensity fluorescence of GFP-RNF8 protein in the damaged areas is higly variable, as described in each inset by the A parameter. The recruitment time (T) varies independently from the intensity fluorescence reached.

Time-lapse imaging permits us to visualize and measure the time evolution of IRIF formation. At time 0, corresponding to the irradiation time, GFP-RNF8 is homogeneously distributed in the cell nucleus. Three minutes after irradiation, the protein starts to accumulate at the damaged areas to form visible IRIF. The fluorescence intensity increases with time over the 30 minutes following irradiation (Fig. 5a). The increase of IRIF fluorescence plotted against the time is illustrated in Fig. 5b for each focus. An exponential fit model permits to measure the IRIF intensity and the protein recruitment time (Supplementary Appendix S3). The IRIF fluorescence intensity varies from 1.3 to twice the fluorescent background value (insets Fig. 5b, parameters A). The recruitment time of the protein (insets Fig. 5b, parameters T) varies from 250 to 700 seconds independently from the A paramenter. These results were reproduced in 28 cells during 3 independent experiments. Using the fit model (described in Supplementary Appendix S3) we measured the GFP-RNF8 Mean ± Standard Deviation recruitment halftime 
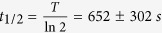
.

## Discussion

We report here the development and validation of an experimental approach that permits, for the first time, the observation of early cellular responses at single α-particle induced DNA damages. This is reached by the use of a microbeam which provides a controlled irradiation at the single-cell level (in time and dose) coupled to online fluorescence imaging.

In comparison to dynamic studies performed with radioactive sources, microbeams have several drawbacks: they are more complex to use and cannot irradiate cells at grazing angles that exploit the full track length. In addition, they require the use of specific dishes containing small volumes of medium restricting image acquisition time to less than an hour in order to avoid excessive cellular stress. Nevertheless, the results reported here demonstrate the ability to deliver single MeV α-particles to cell nuclei with micrometre precision and then subsequently follow GFP-RNF8 IRIF formation over time. The difficulty of detecting MeV α-particles without degrading the microbeam lateral resolution was overcome using thin BNCD membranes with sub-micrometre thicknesses. This solution is similar to that used at GSI Darmstadt for detecting heavy ions^34^,^35^ and was optimized to provide a sufficient signal to count α-particles, for which the electron emission yield is lower. In comparison to the earlier work reported by Fischer *et al*.^34^, BNCD membranes reported here are thinner (400 nm instead of 2 μm) and grown on commercially available Si_3_N_4_ windows facilitating the fabrication and handling. Diamond membranes were actually never used for irradiating living cells as Cesium Iodide (CsI) coated Si_3_N_4_ windows showed more reproducible results^35^.

The BNCD membranes reported here give reproducible detection efficiency and are very homogeneous on millimetre surfaces, corresponding to typical field of views of our fluorescence microscope. In addition to the detection capability, they are also transparent and non-fluorescent, making them compatible with bright field and fluorescence imaging. Over the different experimental runs where these membranes were used, we observed that they were relatively radiation hard and could be used without significant degradation for several days at low beam intensities (i.e. a few thousands particles per second). Another interesting feature is that BNCD membrane can be stored in air without significant degradation. This constitutes a great advantage compared to CsI that showed to be very hygroscopic. Up to now, the fabricated membranes have thicknesses around 400 nm. Since electron emission is a surface phenomenon, thinner detectors could in principle be obtained while maintaining detection efficiency. Thickness reduction could allow smaller spot sizes to be achieved. Further tests to determine the minimum usable thicknesses of BNCD membranes are planned in the future.

The detection of single α-particle traversals allows controlled irradiation of living cells. Post-irradiation analysis shows that GFP-RNF8 accumulates continuously at single α-particle tracks during the first 30 minutes after irradiation. Even if physical interactions and ionizations are confined to less than 100 nm from the core of the tracks, IRIF of 1 to 2 μm in diameter are formed. Their intensity varies from one IRIF to another reflecting most probably the chromatin heterogeneity inside the nucleus (as already reported in previous studies^49^,^50^,^51^). The brightest IRIF shows intensity about two times higher than the undamaged areas. Thus, GFP-RNF8 protein is a useful biological marker which permits to identify and follow over time the regions where DNA DSBs are induced by single α-particles. The knowledge of the irradiation time, necessary for a precise measurement of the evolution of the IRIF over time, is also an important feature of microbeams. Here, irradiation can be achieved with a precision below one second. Finally, the ability to deliver single particles distributed in regular patterns is particularly interesting for the analysis of spatial dynamics of the damaged chromatin. Indeed, in the same cell, the protein recruitment time varies as a function of the hit position in the cell nucleus.

## Materials and Methods

### Membranes preparation

Commercially available Si_3_N_4_ vacuum windows on silicon frame of lateral dimensions 5 × 5 mm, 1 mm squared opening and 150 nm thickness (Silson Ltd., England) were seeded with 5 nm diamond nanoparticles (average size, ADAMAS nano) using electrostatic grafting in PDDAC method (Poly(diallyldimethylammonium chloride))^52^. Boron doped nanocrystalline diamond (BNCD) film growth on nano-seeded Si_3_N_4_ membranes was realized by MWCVD method (Microwave assisted chemical vapour deposition) in home-made ASTEX-type reactor employing trimethylborane gas (TMB) as a source of boron atoms. Following parameters were used during the growth: microwave power 1.2 kW; pressure 40 mbar; methane flow 33 sccm (standard cubic cm per minute); hydrogen flow 100 sccm; TMB flow 10 sccm; growth time 6 h. Synthesized BNCD membranes were employed as-grown with no additional treatment of surface in later experiments.

### Membrane characterisation

The BNCD membranes were characterized using 3 MeV α-particle beams on the AIFIRA facility (Applications Interdisciplinaires des Faisceaux d’Ions en Région Aquitaine)^53^.

#### Electron yield and thickness measurements

The secondary electron emission measurements were performed on the high resolution microbeam line (described previously^54^). The experimental set-up is shown in Fig. 1a. The beam was focused to 0.5 μm FWHM and scanned over a 400 μm × 400 μm area to study the homogeneity of electron emission. The membrane was positioned in the beam path under vacuum and electrons emitted from their surface were collected using a channeltron electron multiplier (CEM, Sjuts^TM^ model KBL15RS/90_H). A 2 mm hole in the head of this particular CEM model was particularly well adapted to enhance the electron collection efficiency while letting the α-particle beam pass through. The α-particles passing through the membrane were detected using a 100 μm thick silicon detector (Canberra, partially depleted detector, 25 mm^2^, 12 keV resolution @ 4.5 MeV) providing a direct measurement of the transmitted energy. All measurements were performed at beam rates between 1000 and 3000 particles per second. Pulse height spectra were obtained using the AIFIRA acquisition system based on a MPA-3 multichannel analyser (FAST ComTec GmbH)^55^.

#### Use of BNCD membranes as a vacuum window and single-ion detector

To validate the use of BNCD membranes as a thin detector for cell targeting experiments, they were fixed as usual vacuum windows on the cell irradiation microbeam line^56^. 3 MeV α-particles were extracted in air through the membranes to irradiate either CR39 track detectors or living cells with single α-particles. The distance between the vacuum window and the sample was approximately 60 μm. The electrons emitted from the BNCD surface in vacuum were collected with the same CEM as the one described previously. Pulses generated by α-particle traversals were counted using a dedicated stand-alone real-time system^55^ triggering a fast electrostatic beam shutter when the required number of particles had been delivered. The *in-air* irradiation configuration is shown in Fig. 2a.

### Solid state track detectors

To ensure the reliability of the dose control as well as the impact of the BNCD membranes on the beam spot size, CR39 solid state track detectors were irradiated in air with single ions. CR39 slabs were positioned at the position of the cell monolayer at a distance of 60 μm from the beam exit window. After irradiation, CR39 were etched in concentrated KOH (12 M, at 80 °C) for 3 minutes. Irradiated patterns were imaged using phase contrast imaging with a Zeiss AxioObserver Z1 microscope (CarlZeiss MicroImaging, GmbH).

### Cell line culture and transfection

HTB96 U2OS cells (from ATCC, CLS, Molsheim) were maintained in McCoy’s 5 A medium (Dutscher) supplemented with 10% (v/v) Fetal Bovine Serum (FBS) and streptomycin/penicillin (100 μg/ml). Cells were kept in a humidified atmosphere at 37 °C and 5% (v/v) CO_2_. A cDNA of human RNF8 inserted into pEGFP-C1 (kindly provided by Jiri Lukas) was used as construct for stable transfections^41^. Viromer Red transfection reagent (Lipocalyx GmbH, Germany) was used for transfections, in combination with the expression vector, according to the manufacturers’ guidelines. Transfected cells were plated 48 h after transfection in presence of different geneticin/G418 dilutions (from 0.1 to 1 mg/ml, GIBCO) were added 72 h after transfection. After 10 days of drug selection, surviving colonies were checked under fluorescence microscopy and GFP-positive colonies were isolated. Several clones were selected and expanded into cell lines for further analysis. Stable expression of recombinant GFP-RNF8 with an exclusive nuclear localization was observed during cultivation for a period of several weeks, indicating a robust growth and reliable expression.

### Immuno-detection

HTB96 U2OS cells were fixed within 1 h after irradiation with paraformaldehyde 4% (w/v) in phosphate-buffered saline (PBS 1X) medium for 15 minutes at room temperature and washed with PBS (pH 7.4, without Ca^2+^ and Mg^2+^). Then, cells permeabilization and saturation were performed using a blocking buffer containing 0.2% (v/v) Triton X-100, 10% (v/v) FBS in PBS for 30 min at room temperature. After three washes with PBS during 5 min, samples were incubated overnight, at 4 °C with anti-human γH2AX rabbit monoclonal antibody (1:1000, 20E3, Cell Signaling). After three more washes with PBS, samples were incubated for 3 h at room temperature with goat anti-rabbit conjugated to Alexa Fluor^488^ antibody (1:2000, Molecular Probes, Invitrogen). Cells were rinsed twice with PBS and nuclei stained with Hoechst^33342^ (1 μM) for 10 min at room temperature. Polypropylene foils were cut and mounted using ProLong Gold Antifade Reagent (Molecular Probes, Invitrogen) overnight at room temperature, and visualized on Zeiss AxioObserver Z1 microscope (CarlZeiss MicroImaging, GmbH).

### Cell irradiations

A custom made support dish is used as described by Bourret *et al*.^56^. This cell dish is adaptable for both ^239^Pu α-source and microbeam irradiation systems. Stably transfected GFP-RNF8 cells were platted on the polypropylene surface (Goodfellow) coated with CellTak (Biosciences) at a density of 14000 cells in 20 μl drop, 24 h before irradiation.

### ^239^Pu (α-source) irradiation

A charged-particle irradiation device, based on a 3.7 kBq ^239^Pu α-source, has been developed for experiments that do not require a precise targeting of individual cells. The α-particles (5105 keV: 12%; 5143 keV: 15%; 5156 keV: 73%) randomly emitted go through 1 mm of air before reaching the polypropylene foil on which cells are attached. The set-up was designed to obtain a few traversals per nucleus for a few minutes of irradiation. During irradiation, cells are maintained in McCoy’s medium. The source was characterized by Monte Carlo simulations, which showed a mean of one traversal per nucleus (1.4 ± 1.1) after 5 min of irradiation, and a mean number of 7.6 ± 3.4 hits per nucleus after 30 min exposure^57^. Cells were exposed to random α-particles irradiation over 30 min, and when the irradiation time was complete, cells were incubated for 30 min at 37 °C to ensure the protein recruitment had been completed in standard culture conditions.

### Microbeam irradiation

α-particles were accelerated by a 3.5 MV electrostatic accelerator (Singletron, High Voltage Engineering Europa, The Netherlands) present in the AIFIRA facility^53^. During microbeam irradiation and image acquisition, cells were maintained in FluoroBrite^TM^ DMEM medium (GIBCO, TermoFisher Scientific) that ensures low background fluorescence. Cells were targeted and irradiated with single α-particles or following different irradiation patterns. Time-lapse imaging was performed online using a 63x objective (LD Plan-Neofluar, NA 0.75, no immersion). Protein re-localization to the damaged area was followed over 30 min. Images were taken every second for the firsts 5 min, then 100 images were taken at 10, 15 and 25 min with a high sensitivity Rolera EM-C^2TM^ Camera (QImaging) using the MicroManager software^58^. Obtained data are corrected for non specific fluorescence bleaching and normalized for the fluorescence intensity measured before irradiation. The accumulation of GFP-RNF8 protein at DSB sites follows a model for a first order step rsponse, previously described by Lukas *et al*.^59^ and illustrated in Supplementary Appendix 3.

## Additional Information

**How to cite this article**: Muggiolu, G. *et al*. Single α-particle irradiation permits real time visualization of RNF8 accumulation at DNA damaged sites. *Sci. Rep.* **7**, 41764; doi: 10.1038/srep41764 (2017).

**Publisher's note:** Springer Nature remains neutral with regard to jurisdictional claims in published maps and institutional affiliations.

## Supplementary Material

Supplementary Dataset
