# Supplementary material for: Single α-particle irradiation permits real-time visualization of RNF8 accumulation at DNA damaged sites
Source: Sci Rep. 2017 Jan 31;7:41764. doi: 10.1038/srep41764 (PMC5282495; doi:10.1038/srep41764)
Supplement: Supplementary Dataset [file srep41764-s1.zip › Muggiolu et al._Suppl Data.doc]

**Single α-particles irradiation permits real-time visualization of RNF8 accumulation at DNA damaged sites**

Giovanna Muggiolu^1,2^, Michal Pomorski^3^, Gérard Claverie^1,2^, Guillaume Berthet^3^, Christine Mer-Calfati^3^, Samuel Saada^3^, Guillaume Devès^1,2^, Marina Simon^1,2^, Hervé Seznec^1,2^ and Philippe Barberet*^1,2^

*1 Université de Bordeaux, Centre d’Etudes Nucléaires Bordeaux Gradignan (CENBG), Chemin du Solarium, 33175 Gradignan, France*

*2 CNRS, UMR5797, Centre d’Etudes Nucléaires Bordeaux Gradignan (CENBG), Chemin du Solarium, 33175 Gradignan, France*

*3 CEA-LIST, Diamond Sensors Laboratory, Gif-sur-Yvette F-91191, France*

Corresponding author: barberet@cenbg.in2p3.fr

**Supplementary material**

**Results**

**Figure S1. Energy loss mapping.** Transmitted energy maps obtained using STIM (Scanning Transmission Ion Microscopy). (a) Map of median energy transmitted through the BNCD membrane. The median energy for each pixel is represented by a grey level showing a good homogeneity of the BNCD thickness. (b) Histogram of the left image showing the dispersion of the transmitted energy on a 400 x 400 μm² area.

**Figure S2. Secondary electron map.** Grey levels represent the number of counts registered by the channeltron at different beam position. The detection efficiency is shown to be very homogeneous on 400 x 400 μm area.

**Apendix S3**

The fit of experimental curves are obtained as follow:

$$Int=1+A\cdot\left(1-e^{-\frac{t-t_{0}}{T}}\right)$$

Where A is the highest intensity value reached; t respresents the time, and t_0_ is the irradiation time fixed to 0 sec; the most relevant parameter is T, which represents the recruitment time expressed in seconds.
